# Supplementary material for: Structural basis of myelin-associated glycoprotein adhesion and signalling
Source: Nat Commun. 2016 Dec 6;7:13584. doi: 10.1038/ncomms13584 (PMC5150538; doi:10.1038/ncomms13584)
Supplement: Supplementary Information — Supplementary Figures 1-19, Supplementary Note 1 and Supplementary References [file ncomms13584-s1.pdf]

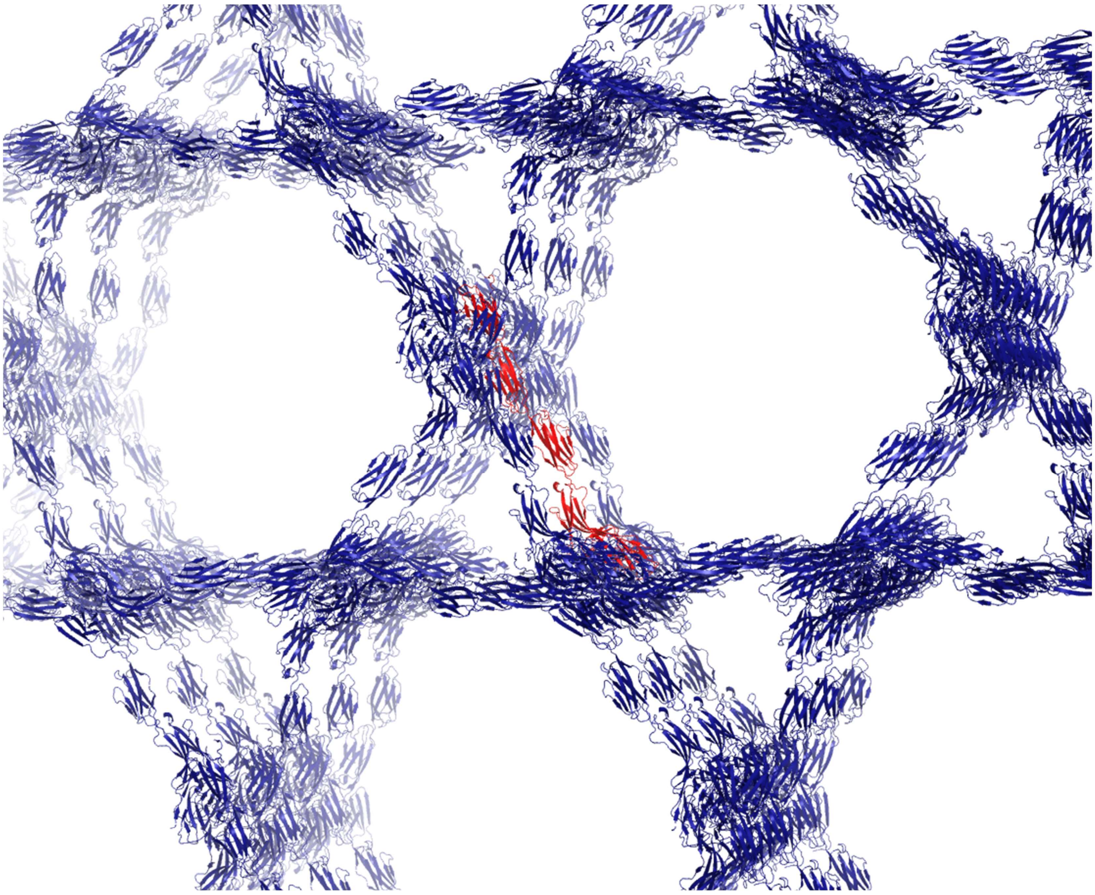

Supplementary Figure 1: High solvent content for MAG_{1-5} deglycosylated crystals

The MAG_{1-5} deglycosylated crystals in space group $P3_22$ have large solvent channels, explaining the high solvent content (91%) for these crystals. The MAG lattice is shown in blue, along the threefold screw axis, with a single MAG monomer highlighted in red.

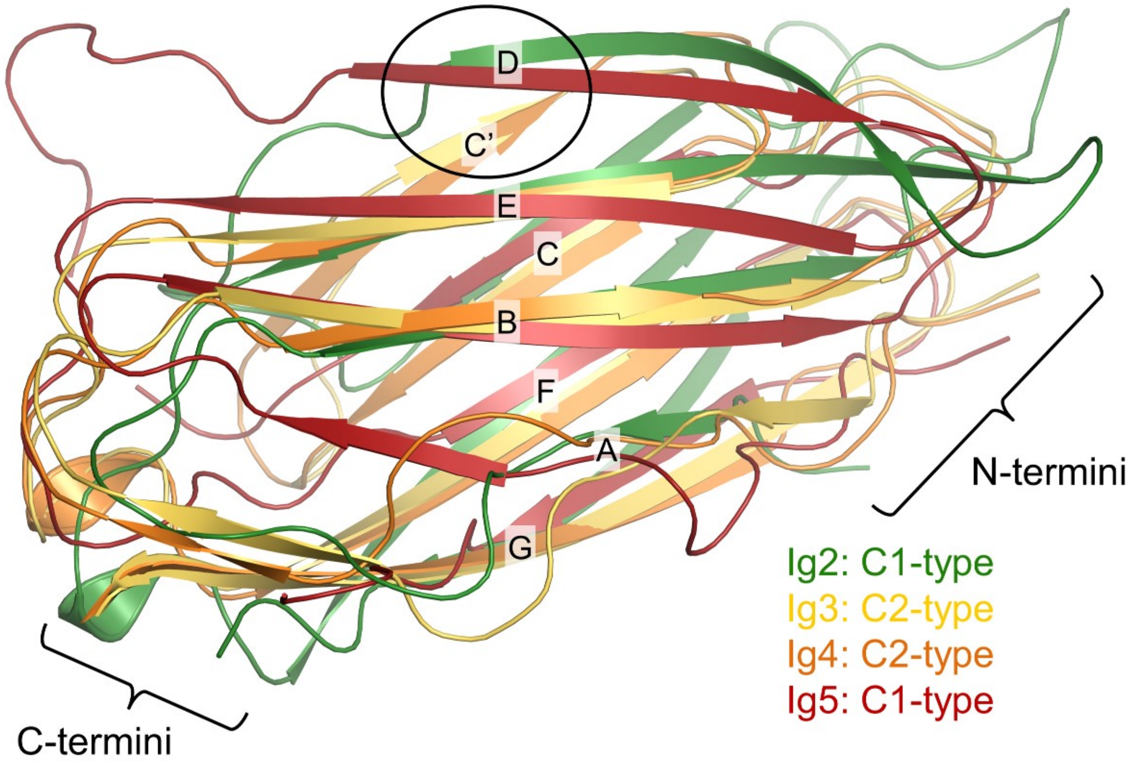

Supplementary Figure 2: Comparison of the MAG Ig2-5 domains, highlighting the different β-strand topology of strand D or C' for the C1- and C2-type Ig folds.

MAG Ig2-5 are all C-type Ig domains, colored as in figure 1a in this figure. Ig3 and Ig4 have a C2-type Ig fold as predicted. Ig2 and Ig5 on the other hand are of the C1-type, contrary to predictions. The C1- and C2-type Ig folds have a different β-strand topology of strand C' and D, indicated by a circle. Whereas the C1-type Ig fold has the D-strand aligning to the ABE sheet (in the frontal sheet from this point of view), this strand is aligning to the bottom CFG sheet in the C2-type fold, where it is referred to as the C'-strand.

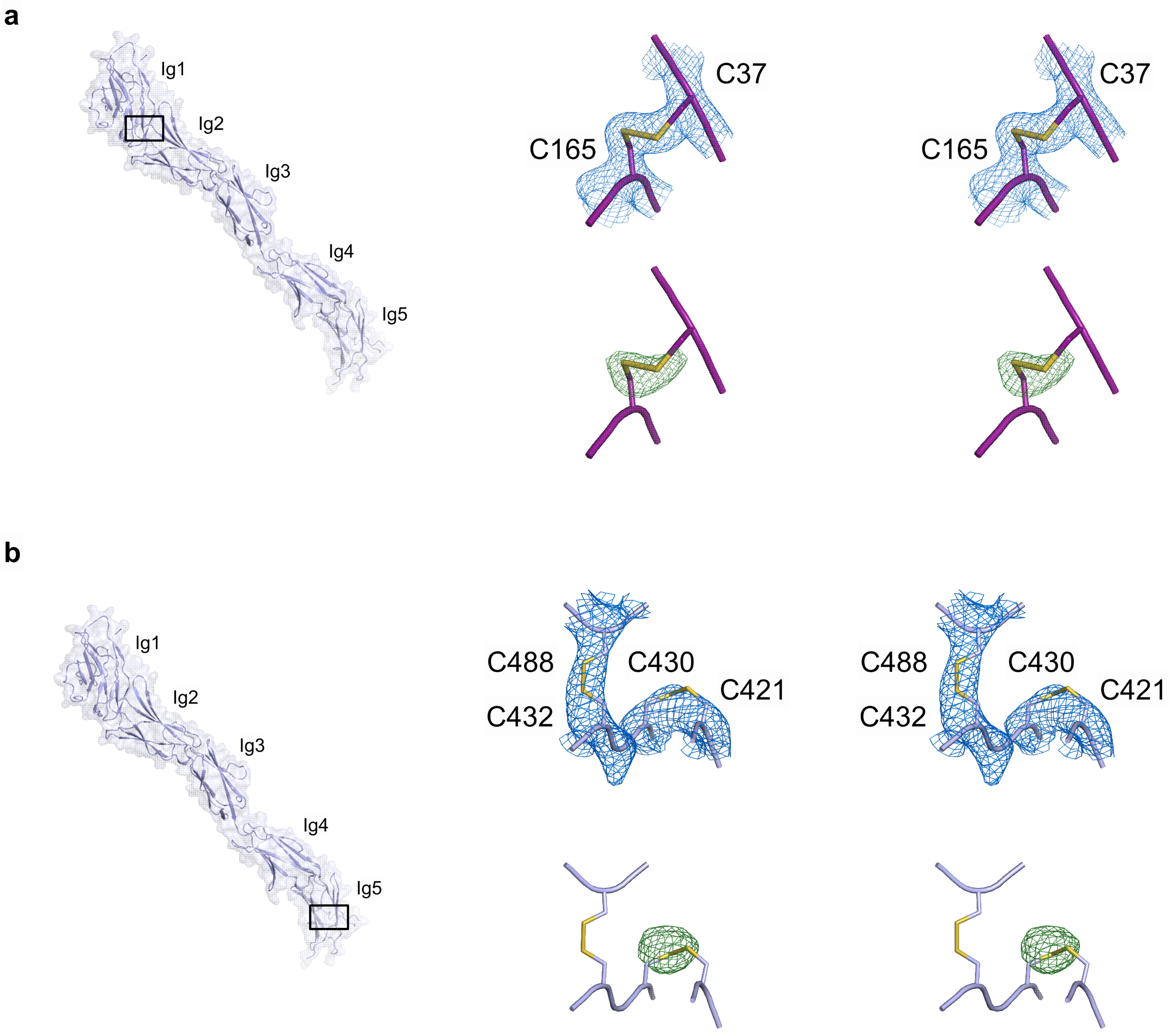

Supplementary Figure 3: Electron density around non-canonical disulfides

(**a**) Stereo image of the electron density around the disulfide of C37-C165 between Ig1 and Ig2 at a contour level of 1.3 σ from the unliganded $MAG_{1\text{-}3}$ structure (top) and simulated annealing omit map of the sulfur atoms of this disulfide at a contour level of 3.5 σ (bottom) (**b**) Stereo image of the electron density around the disulfides of C421-C430 (non-canonical) and the adjacent canonical disulfide C432-C488 of Ig5 at a contour level of 1.2 σ from the deglycosylated $MAG_{1\text{-}5}$ structure (top) and simulated annealing omit map of the sulfur atoms of the C421-C430 disulfide at a contour level of 6 σ (bottom)

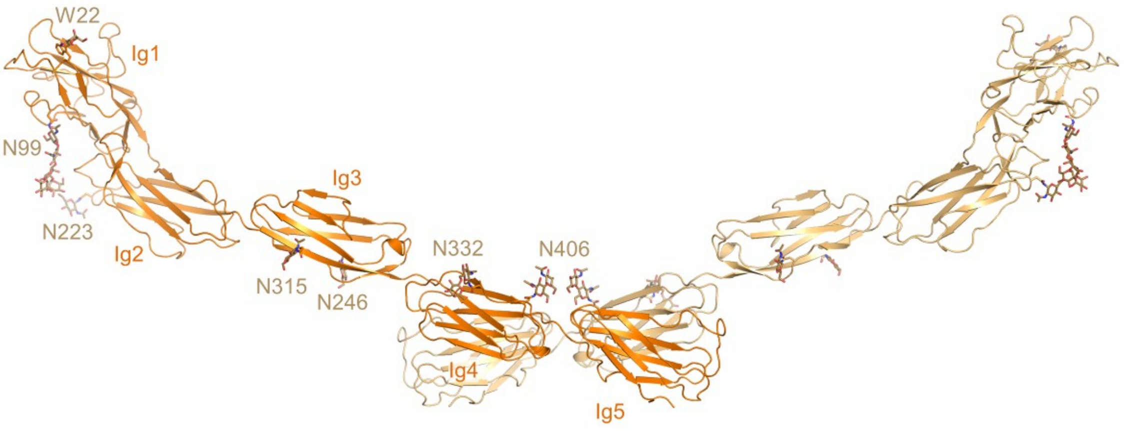

Supplementary Figure 4: Glycosylation of MAG in the lysine-methylated $MAG_{1\text{-}5}$ crystal form

Glycans are shown (sticks) based on the electron density for the lysine-methylated $MAG_{1\text{-}5}$ crystal form (orange, cartoon representation). Numbering for the frontal/left monomer of MAG (darker orange).

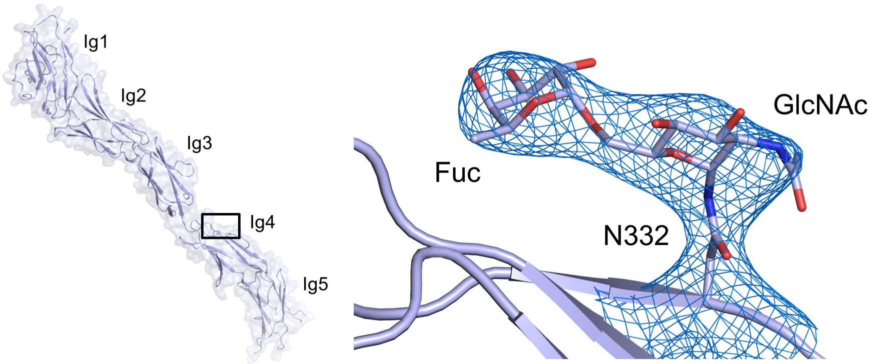

Supplementary Figure 5: Electron density for the fucosylation of the N-linked glycan on N332

The $2F_o{-}F_c$ electron density map contoured at 1.3 σ around N332 is shown for the deglycosylated $MAG_{1\text{-}5}$ crystals, with the N-linked glycan after Endo-H_f treatment modeled (sticks).

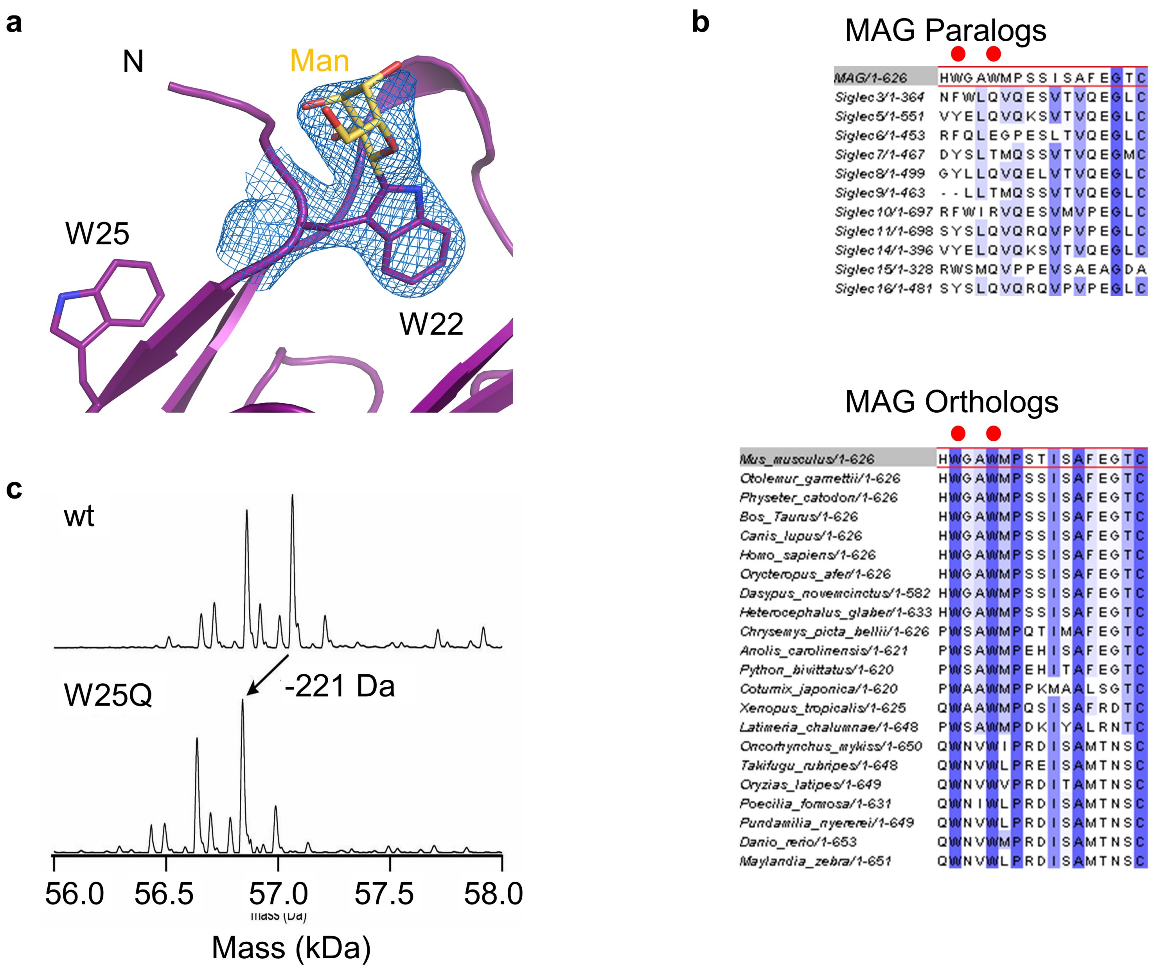

Supplementary Figure 6: Tryptophan mannosylation proximal to the ligand binding site

(**a**) $2F_o{-}F_c$ electron density map contoured at 1.0 σ around W22 in the $MAG_{1\text{-}3}$ unliganded crystals, also showing W25 that is part of the canonical <u>W</u>xxW motif for tryptophan mannosylation. (**b**) Sequence alignments show that the <u>W</u>xxW motif is conserved among MAG orthologs in different vertebrate species, but not in any of the Siglec family paralogs, suggesting functional specificity of this modification for MAG. (**c**) Native mass spectra of $MAG_{1\text{-}5}$ wt and MAG W25Q, showing a mass shift corresponding to the mutation plus loss of the mannosyl group.

Higher-energy Collision Dissociation MS/MS

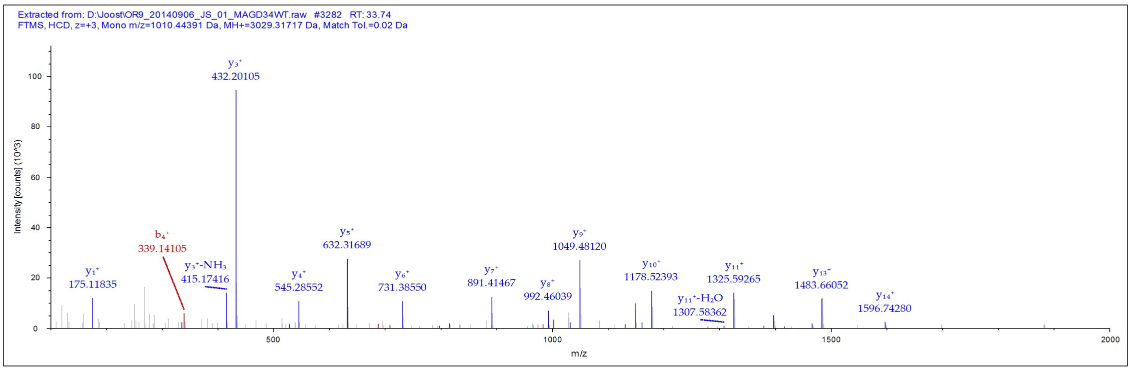

Electron Transfer Dissociation MS/MS

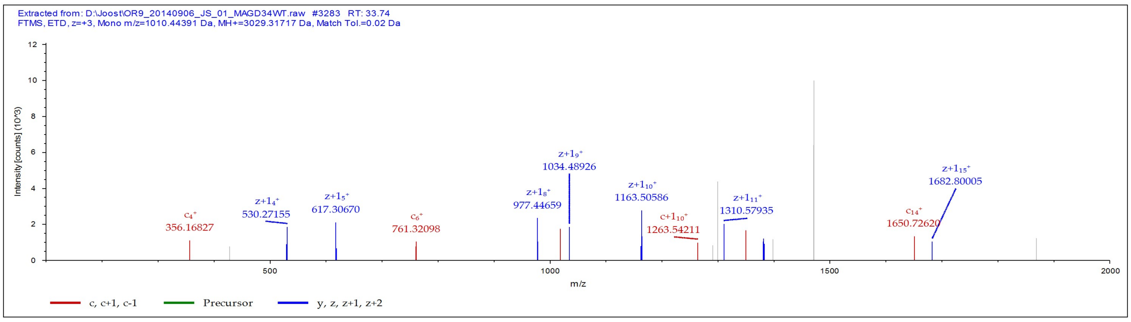

Supplementary Figure 7: Tryptophan-mannosylation is confirmed to be on W22.

MS/MS spectra (by higher-energy collision dissociation or electron transfer dissociation) of the tryptophan-mannosylated N-terminal peptide after trypsinization of wt $MAG_{1\text{-}5}$ show fragment ions corresponding to mannosylation on W22. The sequence was assigned as: GSGHwGAWmPSTISAFEGTcVSIPcR with the following post-translational modifications and adducts: +hexose (tryptophan-mannosylation) on W5, +oxidation of M9, +carbamidomethylation of C20, +carbamidomethylation of C25)

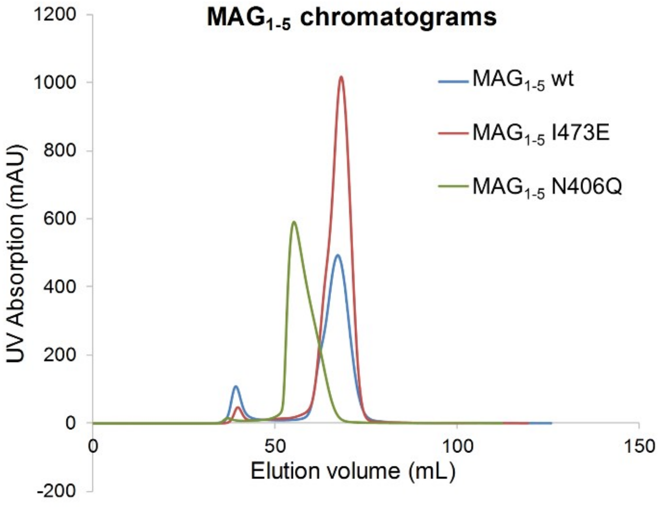

Supplementary Figure 8: Size-exclusion chromatograms of $MAG_{1\text{-}5}$ wt, $MAG_{1\text{-}5}$ I473E and $MAG_{1\text{-}5}$ N406Q

Size exclusion chromatography was performed in large scale as part of the purification procedure on a Hiload 16/60 Superdex200 column as described in the methods section. Absorption was measured at 280 nm. A clear shift to a dimer is observed for the $MAG_{1\text{-}5}$ N406Q mutant.

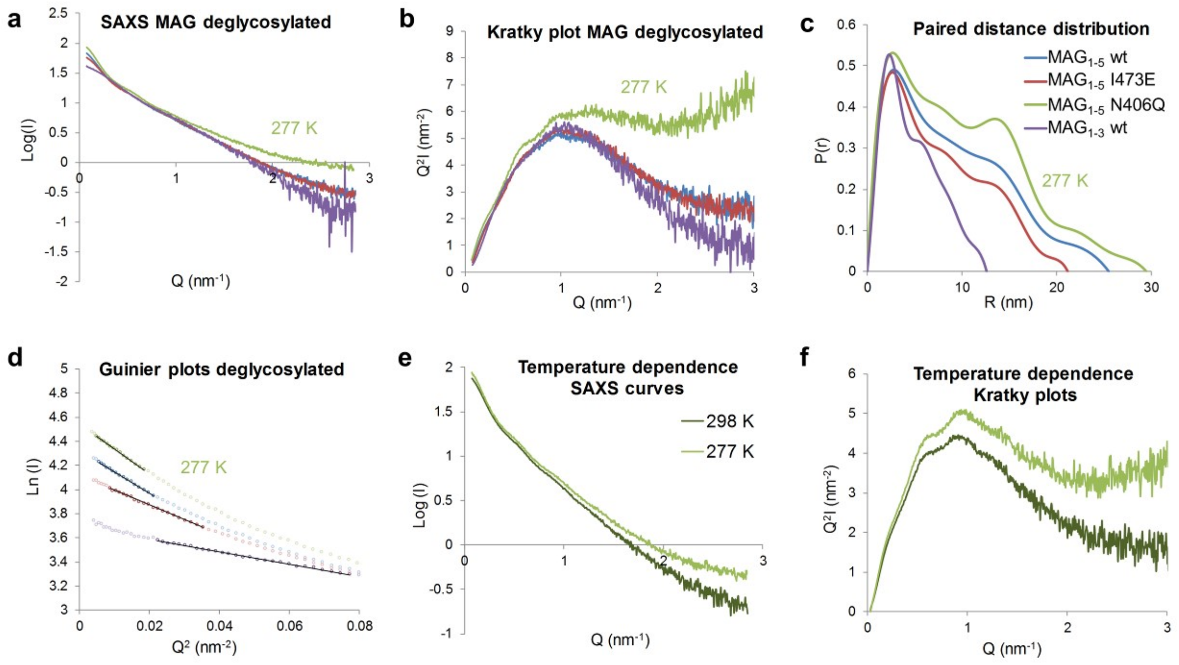

Supplementary Figure 9: SAXS analysis of the deglycosylated MAG variants

(a) Log I vs. Q plot of the different deglycosylated MAG variants. The higher I values at larger scattering angles for the $MAG_{1\text{-}5}$ N406Q variant might be explained by the fact that this was the only construct measured at 277 rather than 293 K (see also panel e), since it was too sensitive to radiation damage and aggregation at 293 K. (b) Kratky plot of the different deglycosylated MAG variants. Again, the $MAG_{1\text{-}5}$ N406Q curve is the only spectrum measured at 277 rather than 293 K (see also panel f). (c) Paired distance distribution function for the different deglycosylated MAG variants. (d) Guinier plots for the different deglycosylated MAG variants. (e) Analysis of the effects of temperature for glycosylated $MAG_{1\text{-}5}$ N406Q at similar concentrations. The Log I vs Q plot is shown, revealing higher scattering intensities (I) for larger scattering angles (Q) when measuring at lower temperatures (277 instead of 293 K). This might explain the similar effect observed for deglycosylated $MAG_{1\text{-}5}$ N406Q in panel a, which was measured at 277 K instead of 293 K. (f) Kratky plot of glycosylated $MAG_{1\text{-}5}$ N406Q at 277 and 293 K, showing a similar effect as seen for the deglycosylated $MAG_{1\text{-}5}$ N406Q in panel b.

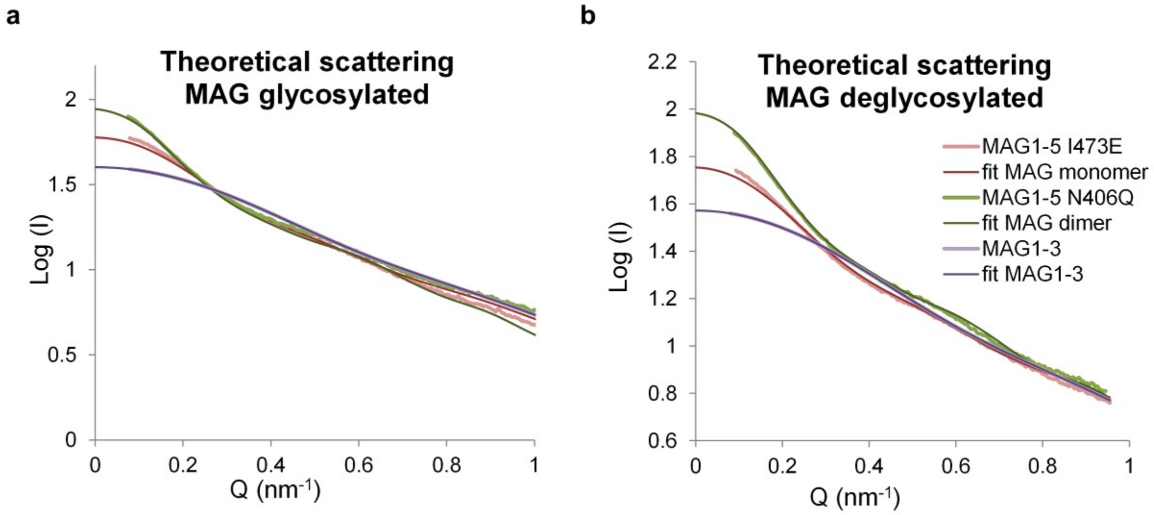

c

Sample	χ
MAG_{1-5} N406Q glycosylated	3.45
MAG_{1-5} I473E glycosylated	2.95
MAG_{1-3} wt glycosylated	2.87
MAG_{1-5} N406Q deglycosylated	8.83
MAG_{1-5} I473E deglycosylated	3.57
MAG_{1-3} wt deglycosylated	1.96

Supplementary Figure 10: Theoretical scattering of crystal structures as determined by CRYSOL compared to experimental SAXS curves

(**a**) Theoretical scattering of the glycosylated crystal forms, calculated using the crystal structure of lysine-methylated MAG_{1-5} in monomeric or dimeric form and of glycosylated MAG_{1-3}, compared to the experimental SAXS curves for the indicated glycosylated MAG forms. Only coordinates of glycans observed in the electron density were used, no extra glycan moieties were modeled. (**b**) Same for the deglycosylated crystal forms, using the crystal structure of the deglycosylated MAG_{1-5} monomer and dimer and of MAG_{1-3} with the glycan coordinates removed, except for the asparagine-attached GlcNAc residues and the tryptophan mannosylation, which are not removed by Endo-H_f deglycosylase. The MAG_{1-3} scattering curves agree very well with the theoretical scattering of the monomeric structures of MAG_{1-3}. (**c**) Table with χ values of fits

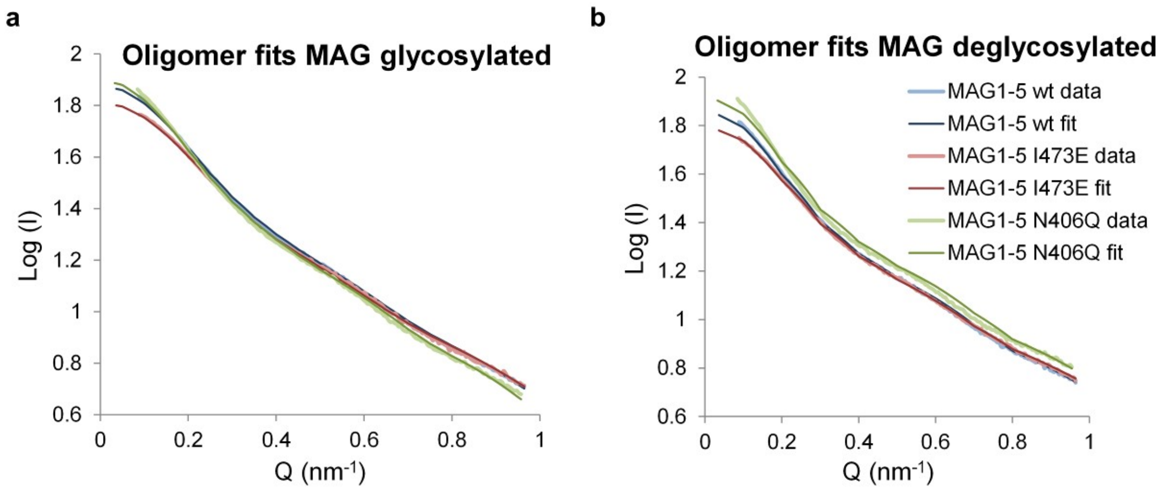

c

Sample	Oligomer Ratio (monomer:dimer)	χ
MAG_{1-5} wt glycosylated	0.72:0.28	4.05
MAG_{1-5} I473E glycosylated	0.92:0.08	1.99
MAG_{1-5} N406Q glycosylated	0.49:0.52	3.95
MAG_{1-5} wt deglycosylated	0.50:0.50	3.72
MAG_{1-5} I473E deglycosylated	0.76:0.24	3.62
MAG_{1-5} N406Q deglycosylated	0.46:0.54	9.92

Supplementary Figure 11: OLIGOMER fits to experimental SAXS curves for MAG_{1-5} wt and mutants

OLIGOMER fits to SAXS curves of glycosylated (**a**) and deglycosylated (**b**) MAG_{1-5}, using the same models as in Supplementary Fig. 10. (**c**) Monomer-dimer ratios determined by OLIGOMER and χ of the fits .

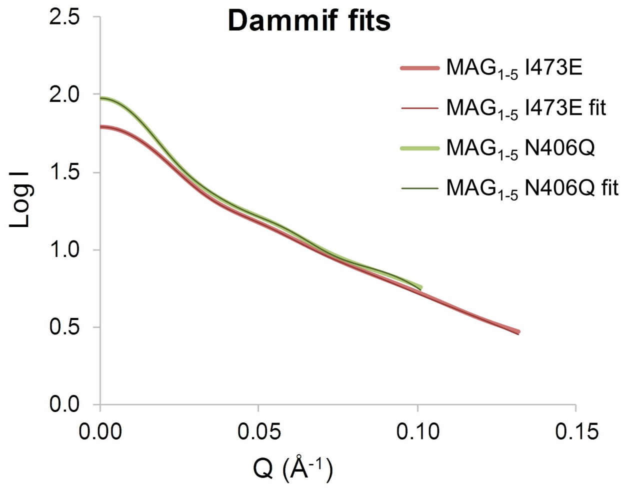

Supplementary Figure 12: Fits of Dammif *ab-initio* modeling to the SAXS data of deglycosylated MAG_{1-5} mutants

Dammif fits corresponding to the bead models shown in figure 2g, corresponding to the data of deglycosylated MAG_{1-5} I473E (χ^2=1.05) and MAG_{1-5} N406Q (χ^2=1.33). Twofold rotational symmetry was enforced for the MAG_{1-5} N406Q model.

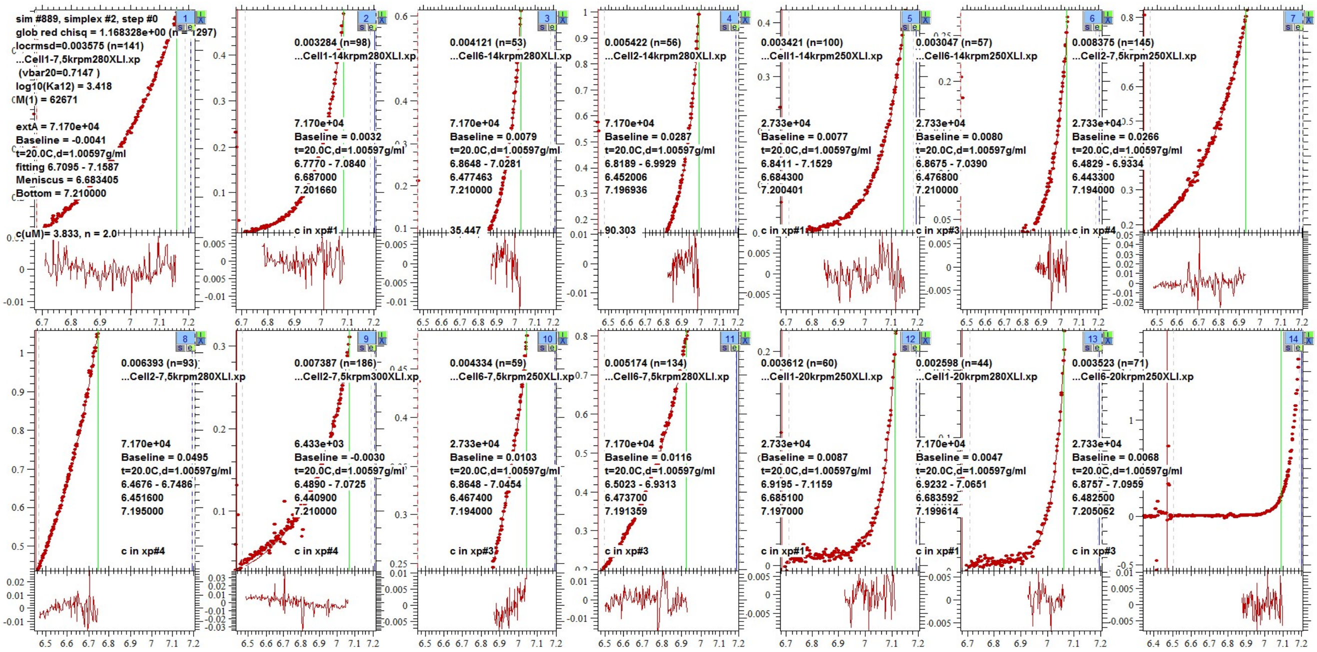

Supplementary Figure 13: SEDPHAT analysis of se-AUC data for $MAG_{1\text{-}5}$ wt glycosylated

A global analysis was performed using different concentrations and different rotation speeds, in which MAG was modeled as a monomer-dimer equilibrium with the M_m floated. Rotation speed can be read from the second line in each panel (7.5, 14 or 20 krpm) and concentration in µM from the bottom line (after c) in each panel. In the panels beneath the curves the residuals from the fit are shown.

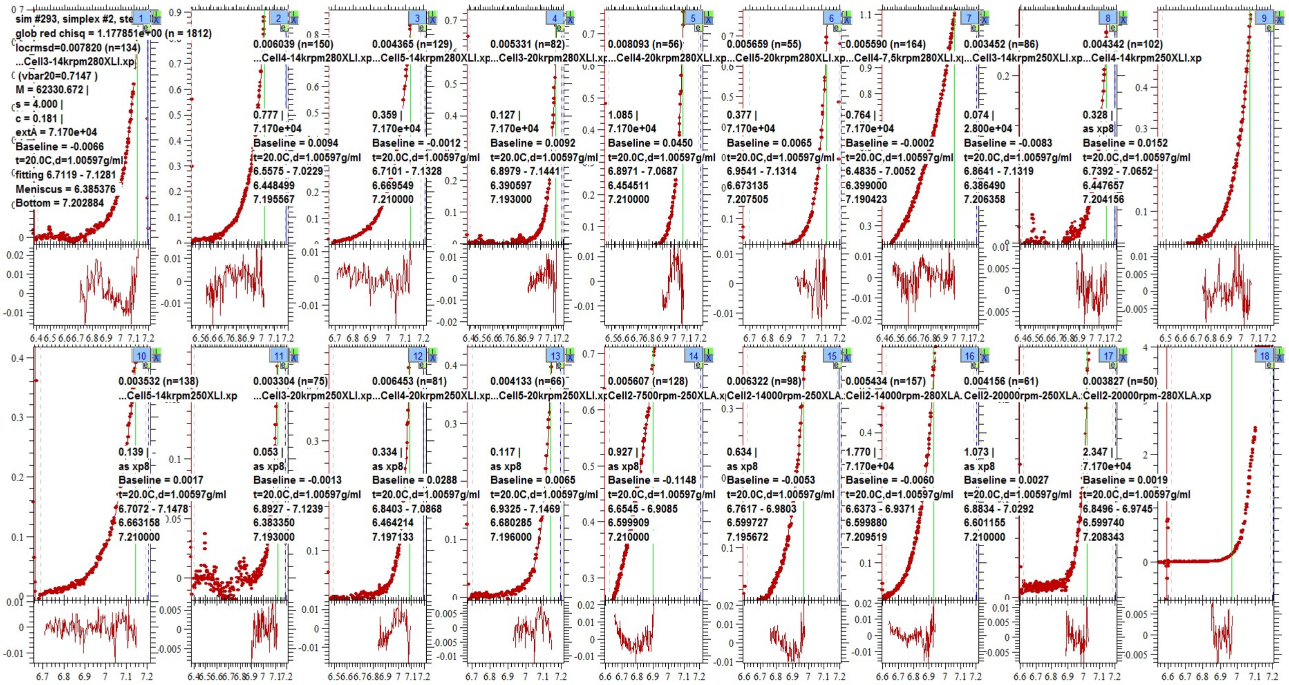

Supplementary Figure 14: SEDPHAT analysis of se-AUC data for $MAG_{1\text{-}5}$ I473E glycosylated

Same as Supplementary Fig. 13, but modeled as a single species, M_m floated

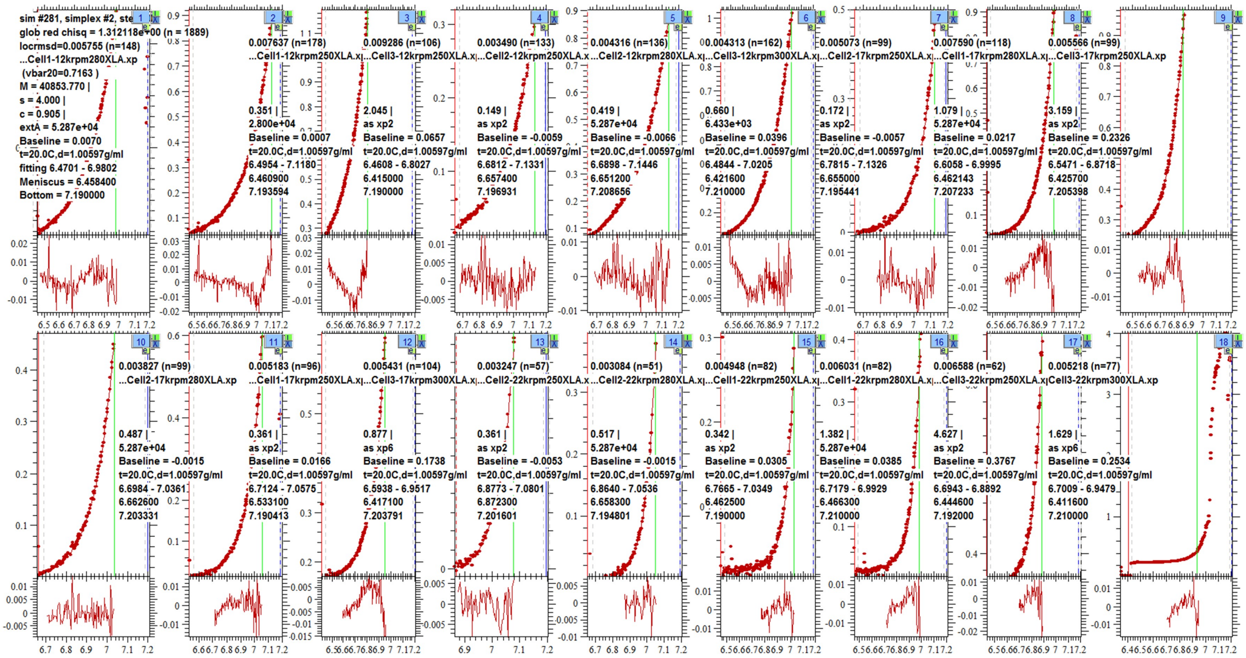

Supplementary Figure 15: SEDPHAT analysis of se-AUC data for $MAG_{1\text{-}3}$ wt glycosylated

Same as Supplementary. Fig. 13, but modeled as a single species, M_m floated

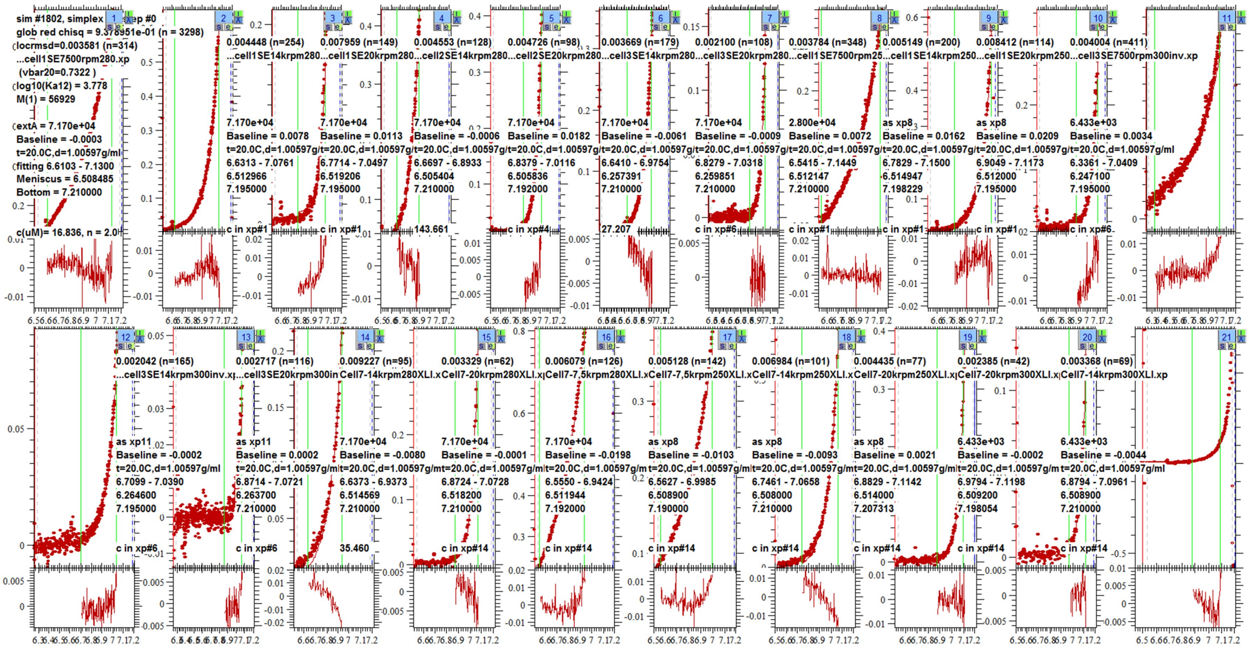

Supplementary figure 16: SEDPHAT analysis of se-AUC data for $MAG_{1\text{-}5}$ wt deglycosylated

Same as Supplementary Fig. 13, but modeled as a monomer-dimer equilibrium, M_m fixed

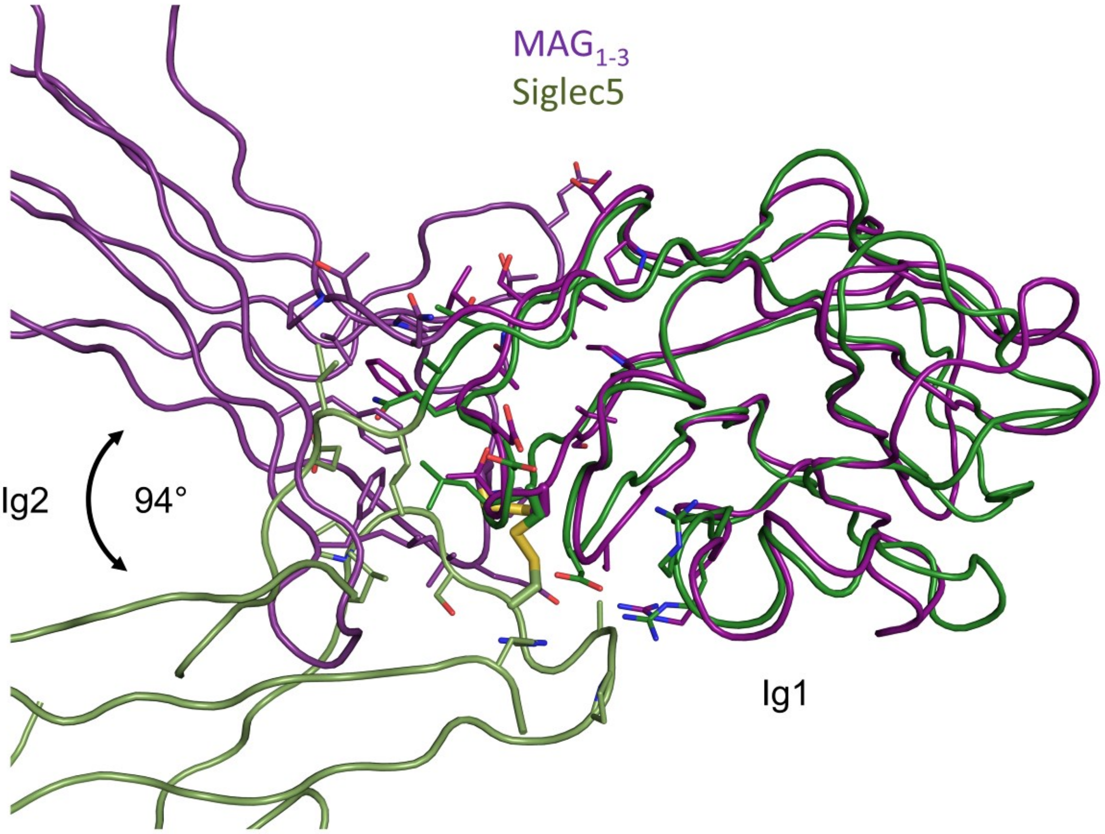

Supplementary Figure 17: Comparison of the interdomain orientation between the two N-terminal domains of MAG and siglec5

Siglec5 is the only other siglec family member for which a structure is available comprising more than one domain. Comparison of the MAG_{1-3} (purple) and siglec5 (green) structures, aligned by their N-terminal V-type Ig domains, reveals a marked domain rotation of 94° of the second Ig domain (light) with respect to the first Ig domain (dark). Both have a conserved interdomain disulfide between the first two domains (shown as thick sticks), as do most other siglec family members. The domain rotation is probably the result of variations of the other residues at the interdomain interface (shown as sticks) as well as differences in the structure of the second domain.

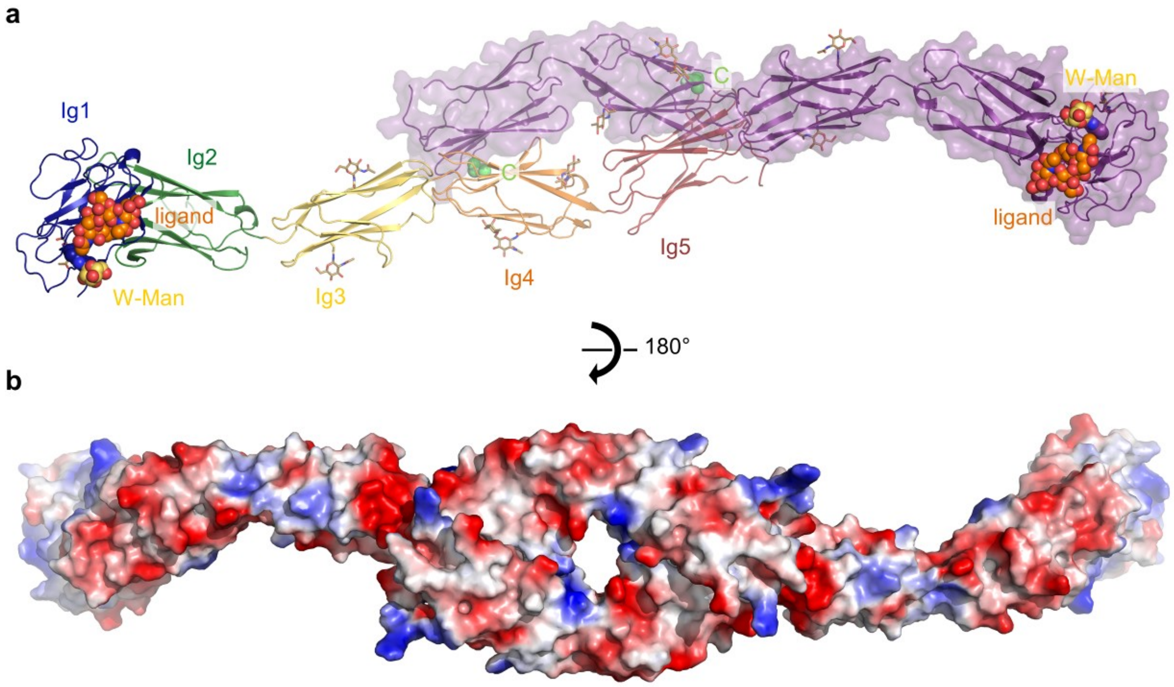

Supplementary Figure 18: Top and bottom views of the MAG dimer

(**a**) Top view of deglycosylated $MAG_{1\text{-}5}$ dimer (representation as in Fig. 5, but rotated 90 °), one molecule colored by domain and the other purple with the surface shown. The co-purified carbohydrate ligand (orange spheres), the tryptophan mannosylation (yellow spheres, labelled W-Man), the other glycosylation sites (brown sticks) and the membrane-proximal C-termini (green spheres) are indicated. (**b**) Bottom view of deglycosylated $MAG_{1\text{-}5}$ dimer with electrostatic surface potential shown (red is negatively charged, blue is positive, white is neutral).

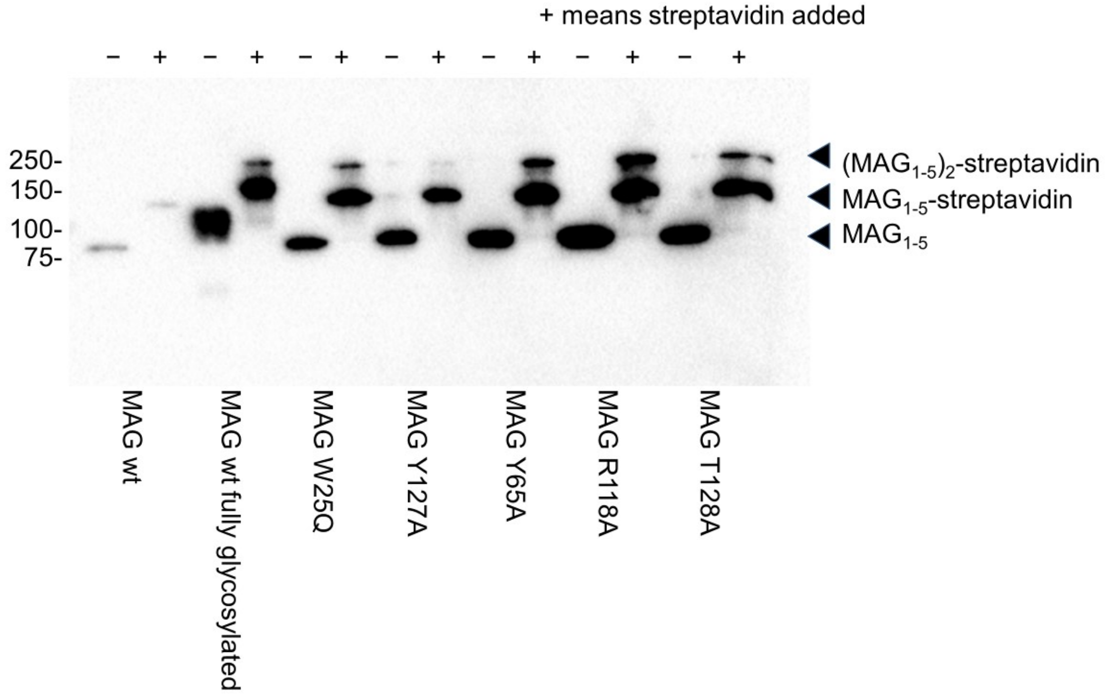

Supplementary Figure 19: Gel-shift assay confirms biotinylation of MAG mutants as ligands for GT1b ganglioside liposome SPR.

The different binding-site mutants were expressed with a biotin acceptor peptide (BAP) and His_6-tag and co-expressed with BirA biotin transferase in HEK cells as described in the Methods section. After small-scale Ni-affinity purification, biotinylation was confirmed by a gel-shift after addition of an excess of streptavidin. The second sample is the same construct as the first, but expressed in HEK cells without the GnTI−/−, thus having mature N-linked glycans, but this construct was not used for further analyses. All samples appear fully biotinylated as the lower band completely disappears, the extra top band that appears sometimes for the samples with streptavidin likely corresponds to two biotinylated MAG proteins binding to a single streptavidin tetramer.

Supplementary notes

Comparison of Siglec structures

We present the first structure of a full extracellular domain of a Siglec family member. All other Siglecs function in the immune system rather than in the nervous system, but they share an N-terminal V-type Ig domain for recognizing sialic acid moieties, followed by 1 to 16 C-type Ig domains[1].

Other available Siglec structures comprise either only the N-terminal V-type Ig (Siglec1/Sialoadhesin[2] and Siglec7[3]), or the two N-terminal domains (Siglec5[4]). Comparison of the two N-terminal domains of Siglec5 with those of MAG reveals a markedly different interdomain orientation (rotation of 94°, Supplementary Fig. 17). This is surprising, since Siglec5 and MAG, as well as most other Siglecs, have a conserved disulfide bond between the two N-terminal domains. This domain rotation is probably caused by differences in the other amino acids at the interdomain interface between Ig1 and Ig2, as well as a structurally less conserved second Ig domain (Supplementary Fig. 17).

Siglec10 and Siglec11, like MAG, have five Ig domains in total. Whether they can form dimers in a similar fashion to MAG and whether this is related to their function, remains to be investigated.

Additional insights into ligand recognition

One of the two co-purified tetrasaccharide ligands of MAG has a glycolyl variant of sialic acid (Neu5Gc instead of Neu5Ac), that is not biosynthesized by humans and likely originated from the beef digest Primatone that was part of the expression medium. The fact that this low-abundant sugar is prevalent in purified MAG sample suggests that it binds stronger than the N-acetylneuraminic acid variant. This could be explained by an extra hydrogen bond that the

hydroxyl group of the glycolyl variant could form with the side chain of conserved residue N125 in MAG. However, this is in conflict with studies showing no binding of MAG to Neu5Gc-containing gangliosides[5,6]. Also it is interesting that MAG seems to have a preference for tetrasaccharides rather than trisaccharides, even though no density is observed for the fourth sugar in the deglycosylated MAG_{1-5} crystal form. These observations are relevant for rational drug design efforts, as the ganglioside binding site is an intensively studied drug target[7].

Glycosylation on Asparagine 406 may serve a regulatory function

We show that the N-linked glycan on N406, that appears to clash with its symmetry-related dimerization partner, reduces dimerization potential in solution. This suggests that the N406 glycosylation site may serve to regulate self-interaction and thereby myelination. For example, MAG can carry HNK-1 containing N-linked glycans on N406, which have a terminal negatively charged glucuronic acid[8] that could potentially modulate MAG dimerization by coulombic repulsion. Regulation may occur at the biosynthesis level, in which the expression levels of glycosylation enzymes in the secretory pathway vary during development. Alternatively, extracellular trimming by glycosidase enzymes may spatially and temporally regulate MAG dimerization by acting on N406. A putative candidate glycosidase for such a role is the antiaging factor Klotho, a secreted glycosidase present in the cerebrospinal fluid[9,10]. It is known to have β-glucuronidase activity and to trim N-linked glycans of neuronal membrane-anchored proteins[10,11]. Trimming of these charged glucuronic acid groups from the N-linked glycans on MAG N406 by Klotho could reduce the coulombic repulsion and thus enhance MAG dimerization.

In line with this hypothesis, Klotho knockout mice have an impaired myelination phenotype[12]. Furthermore, it was shown that downstream intracellular targets of Klotho in oligodendrocyte precursor cells (OPCs) are part of the Akt and Erk signaling pathways[12]. These pathways are also downstream of Fyn kinase in OPCs and oligodendrocytes[13], suggesting that MAG/Fyn could be a transmembrane link connecting extracellular Klotho activity with intracellular signaling events in OPCs. We speculate that in healthy individuals, Klotho could be involved in trimming glucuronic acid-containing N-linked glycans on N406 of MAG, thus regulating MAG dimerization, Fyn signaling and thereby myelination.

Supplementary References

1. Crocker, P. R., Paulson, J. C. & Varki, A. Siglecs and their roles in the immune system. *Nat. Rev. Immunol.* **7,** 255–266 (2007).

2. May, A. P., Robinson, R. C., Vinson, M., Crocker, P. R. & Jones, E. Y. Crystal structure of the N-terminal domain of sialoadhesin in complex with 3' sialyllactose at 1.85 A resolution. *Mol. Cell* **1,** 719–728 (1998).

3. Alphey, M. S., Attrill, H., Crocker, P. R. & van Aalten, D. M. F. High resolution crystal structures of Siglec-7. Insights into ligand specificity in the Siglec family. *J. Biol. Chem.* **278,** 3372–7 (2003).

4. Zhuravleva, M. a., Trandem, K. & Sun, P. D. Structural Implications of Siglec-5-Mediated Sialoglycan Recognition. *J. Mol. Biol.* **375,** 437–447 (2008).

5. Collins, B. E. *et al.* Sialic acid specificity of myelin-associated glycoprotein binding. *J. Biol. Chem.* **272,** 1248–55 (1997).

6. Collins, B. E., Fralich, T. J., Itonori, S., Ichikawa, Y. & Schnaar, R. L. Conversion of

cellular sialic acid expression from N-acetyl- to N-glycolylneuraminic acid using a synthetic precursor, N-glycolylmannosamine pentaacetate: inhibition of myelin-associated glycoprotein binding to neural cells. *Glycobiology* **10,** 11–20 (2000).

7. Schwardt, O., Kelm, S. & Ernst, B. in *Topics in Current Chemistry* 151–200 (Springer Verlag, 2013). doi:10.1007/128_2013_498

8. Burger, D., Pidoux, L. & Steck, A. J. Identification of the glycosylated sequons of human myelin-associated glycoprotein. *Biochem. Biophys. Res.* **197,** 457–464 (1993).

9. Li, S.-A. *et al.* Immunohistochemical localization of Klotho protein in brain, kidney, and reproductive organs of mice. *Cell Struct. Funct.* **29,** 91–99 (2004).

10. Tohyama, O. *et al.* Klotho Is a Novel β-Glucuronidase Capable of Hydrolyzing Steroid β-Glucuronides. *J. Biol. Chem.* **279,** 9777–9784 (2004).

11. Chang, Q. *et al.* The beta-glucuronidase klotho hydrolyzes and activates the TRPV5 channel. *Science* **310,** 490–493 (2005).

12. Chen, C.-D. *et al.* The antiaging protein Klotho enhances oligodendrocyte maturation and myelination of the CNS. *J. Neurosci.* **33,** 1927–39 (2013).

13. Taveggia, C., Feltri, M. L. & Wrabetz, L. Signals to promote myelin formation and repair. *Nat. Rev. Neurol.* **6,** 276–87 (2010).
